# Establishment and validation of post-PCI nomogram in elderly patients with acute coronary syndromes

**DOI:** 10.3389/fcvm.2025.1529476

**Published:** 2025-03-07

**Authors:** Xing-Yu Zhu, Zhi-Meng Jiang, Xiao Li, Fei-Fei Su, Jian-Wei Tian

**Affiliations:** ^1^ Graduate School of Hebei North University, Zhangjiakou, Hebei, China; ^2^ Department of Cardiovascular Medicine, Air Force Medical Center, Chinese People’s Liberation Army, Beijing, China

**Keywords:** acute coronary syndromes, major adverse cardiovascular events, percutaneous coronary intervention, nomogram, least absolute shrinkage selection operator

## Abstract

**Objective:**

The objective of this study was to create and validate a clinical prediction model for the incidence of major adverse cardiovascular events (MACE) within one year after percutaneous coronary intervention (PCI) in elderly patients diagnosed with acute coronary syndromes (ACS)

**Methods:**

The study will use 70% of the 738 patients for model training and the remaining 30% for model validation. The feature recursive elimination algorithm (RFE) and the least absolute shrinkage selection operator (LASSO) regression technique will be used to identify the best combination of features. We compare the clinical prediction model we constructed with GRACE in terms of discrimination, calibration, recall, and clinical impact

**Results:**

We used the RFE and LASSO regression technique to select 8 key variables from 44 candidates for our predictive model. The predictive model was found to have a good fit based on the Hosmer-Lemeshow test results (*χ*^2^ = 6.245). Additionally, the Brier score of the clinical prediction model was 0.1502, confirming its accuracy. When comparing our clinical prediction model to the widely used GRACE scoring system, the results showed that our model had slightly better predictive efficacy for the dataset involved in this study. The NRI was 0.6166, NRI + was 0.2262, NRI- was 0.3904, and IDI was 0.1272, with a *P* value of <0.001. The validation set's AUC was 0.787, indicating the prediction model has high differentiation and discriminative ability.

**Conclusion:**

This model assists in the early identification of the risk of MACE within one year after PCI for ACS in elderly patients.

## Introduction

Acute coronary syndrome (ACS) refers to the acute myocardial ischemic conditions that result from the rupture or erosion of unstable atherosclerotic plaques in the coronary arteries, leading to the formation of fresh thrombi (1, 2). It is estimated that over 7 million individuals worldwide are diagnosed with ACS annually (2). ACS is one of the leading causes of morbidity and mortality among patients (3). Despite substantial advancements in percutaneous coronary intervention (PCI), a significant number of patients continue to suffer from major adverse cardiovascular events (MACE) each year. This issue is particularly pronounced within the elderly population, underscoring the critical nature of the problem (4–6). The frequent occurrence of MACE not only poses a serious threat to patients’ lives but also substantially elevates their financial burden. Therefore, it is imperative to conduct early comprehensive assessments and predictions for elderly ACS patients undergoing PCI, aimed at effectively managing risk factors and minimizing the incidence of MACE (7). Nonetheless, prognostic research concerning post-PCI outcomes in elderly ACS patients remains relatively scarce in China.

Clinical prediction models are multifactorial models that estimate the probability of having a disease or the likelihood of an outcome occurring in the future (8). Prognostic modeling is a type of modeling that focuses on predicting the probability of future outcomes, such as disease recurrence, death, disability, and the development of complications, based on the current disease state (9). Current prediction models for ACS, such as the GRACE score, have demonstrated utility in predicting short-term mortality and recurrent ischemic events in mixed-age populations (10, 11). However, these models may lack precision when applied to elderly patients, who often present with unique clinical features, comorbidities, and frailty. Additionally, the existing models focus largely on short-term risk, neglecting long-term outcomes such as MACE following PCI. This highlights a critical gap in addressing the specific prognostic needs of elderly ACS patients in clinical practice.

The present study aims to address the limitations of existing research in this field by developing and validating a clinical prognostic model tailored to predicting the 1-year risk of major adverse cardiovascular events (MACE) post-percutaneous coronary intervention (PCI) in elderly patients with acute coronary syndrome (ACS). The model utilizes machine learning techniques to identify key predictive factors, to improve the accuracy of risk stratification. This, in turn, should assist clinicians in optimizing management strategies and personalizing treatment plans. The ultimate goal of this study is to enhance clinical decision-making and reduce the burden of adverse cardiovascular events in this high-risk population.

### Information and methodology

#### Study subjects and subgroups

This study was approved by the Ethics Committee of the Chinese People's Liberation Army Air Force Medical Center. All research methods adhered to the Declaration of Helsinki, as well as relevant guidelines and regulations. This retrospective, observational, single-center study analyzed medical data from 1,159 patients diagnosed with acute coronary syndrome (ACS) who underwent PCI between October 1, 2019, and January 1, 2023, in the Department of Cardiovascular Medicine at the Air Force Specialty Medical Center. Based on inclusion and exclusion criteria, 738 patients were selected and divided into a training set (511 patients) and a validation set (227 patients) in a 7:3 ratio. Subsequently, patients were categorized into MACE and non-MACE groups depending on the occurrence of MACE within one year.

##### Inclusion criteria

(1) Age ≥60 years; (2) Patients undergoing treatment for ACS in conjunction with PCI. Refer to ACS for the diagnostic criteria of Acute Coronary Syndromes (1). (3) The MACE criteria comprises recurrent angina, restenosis, cardiac death, acute myocardial infarction, and rehospitalization due to cardiovascular causes (such as unstable angina, severe arrhythmia, heart failure, etc.). (4) Complete clinical case information.

##### Exclusion criteria

(1) Inadequate information on relevant cases: Patients with severe hepatic or renal dysfunction, hematological disorders, infectious diseases, malignancies, autoimmune diseases, or inflammatory conditions were excluded. This exclusion applied to any patient meeting any of the above criteria.

## Research methodology

### Basic information collection

Collect the patient's basic clinical characteristics, including age, gender, height, weight, admission blood pressure, resting heart rate, and history of smoking and alcohol use. Gather details of the patient's medical history, such as hyperlipidemia, hypertension, diabetes mellitus, peripheral vascular disease, and cerebrovascular disease. Auxiliary examinations include coronary angiography to assess the location and severity of stenosis in the coronary arteries and the number of affected branches. A cardiac ultrasound is performed to evaluate the left ventricular ejection fraction (LVEF). Blood tests are conducted to analyze white blood cell count, neutrophils, lymphocytes, hemoglobin, platelets, ultrasensitive C-reactive protein, coagulation profile, liver and kidney function, cardiac biomarkers, blood lipids, and electrolyte levels. The Prognostic Nutritional Index (PNI) evaluates nutritional status using the formula: PNI = albumin (g/L) + 5 × lymphocytes (×10⁹ /L), as described in previous studies. The GRACE score, a predictor of ACS outcomes, was also calculated based on established methodologies from prior research (12).

### Telephone follow-up visit

The endpoint event for follow-up was the occurrence of a major adverse cardiovascular event within one year. Follow-up started on 1st October 2019 and ended on 1st January 2024. Any major adverse events that occurred after the patient's discharge were recorded.

### Statistical methods

In-depth statistical analyses were performed using R4.2.3 and SPSS 27.0. Continuous variables with a normal distribution were expressed as mean ± standard deviation ($\bar{x}\pm s$) and compared using t-tests, while non-normally distributed variables were expressed as median and interquartile range [M(Q1, Q3)] and compared using the Mann–Whitney U test. Categorical variables were presented as percentages and analyzed with the *χ*^2^ test. To avoid overfitting and multicollinearity, the training dataset was processed using the feature recursive elimination algorithm and LASSO regression analysis to identify key factors contributing to MACE in elderly ACS patients within one year of PCI. Pearson correlation analysis was conducted to examine the relationships between selected variables and MACE occurrence. Multifactorial logistic regression was used to develop clinical prediction models and Nomograms, with model accuracy assessed using the Brier score. Model performance was validated using the Hosmer-Lemeshow test for goodness-of-fit (chi-squared statistic and *P*-value), calibration curves, and the area under the ROC curve (AUC). Decision curve analysis (DCA) and clinical impact curve (CIC) were used to evaluate the model's practical utility. Statistical significance was set at *P* < 0.05. Finally, the predictive performance of the newly developed clinical models was compared to GRACE scores using the integrated discrimination improvement (IDI) and net reclassification improvement (NRI) metrics.

## Results

### Training set clinical data analysis

A retrospective collection of 1,159 consecutive cases diagnosed with acute coronary syndrome (ACS) who underwent PCI between 1 October 2019 and 1 January 2023 was conducted at the Air Force Specialty Medical Centre. Based on the inclusion and exclusion criteria, 738 cases were included. These were then randomly divided into a training set of 511 cases and a validation set of 227 cases, in a 7:3 ratio. The model was built using the training set and tested using the validation set. The study divided ACS patients into two groups: those who experienced MACE within one year (MACE group) and those who did not (non-MACE group). Table 1 presents the baseline characteristics of these groups in the training set.

**Table 1:** Clinical data comparison in training Set.

Variables	All patients (*N* = 511)	Non-MACE (*N* = 374)	MACE (*N* = 137)	*P* values
Unstable angina, *n* (%)	373 (73)	287 (77)	86 (63)	0.002
NSTEMI, *n* (%)	44 (9)	30 (8)	14 (10)	0.544
STEMI, *n* (%)	94 (18)	57 (15)	37 (27)	0.004
Dyslipidemia, *n* (%)	297 (58)	230 (61)	67 (49)	0.014
Hypertension, *n* (%)	353 (69)	255 (68)	98 (72)	0.537
Smoking, *n* (%)	81 (16)	60 (16)	21 (15)	0.953
Diabetes mellitus, *n* (%)	209 (41)	154 (41)	55 (40)	0.914
Female, *n* (%)	366 (72)	274 (73)	92 (67)	0.213
Age, M (Q1, Q3)	69 (64,75)	68 (64,73)	73 (67,80)	<0.001
Heart. Rate, M (Q1, Q3)	76 (68,85)	76 (68,85)	76 (68,86)	0.663
SBP, M (Q1, Q3)	130 (120,141)	129 (120,140)	130 (118,143)	0.519
DBP, M (Q1, Q3)	75 (67,85)	75 (67,85)	74 (66,84)	0.288
Height, M (Q1, Q3)	1.68 (1.62,1.72)	1.69 (1.62,1.73)	1.68 (1.6,1.7)	0.014
Weight, M (Q1, Q3)	70 (62,77)	70 (62.5,78.75)	70 (60,75)	0.065
BMI, Mean ± SD	25 ± 3.12	25.07 ± 3.16	24.82 ± 3.03	0.415
leucocyte, [×10^9 ^/L, M (Q1, Q3)]	6.62 (5.52,8.3)	6.62 (5.44,8.28)	6.7 (5.7,8.9)	0.288
neutrophil, [×10^9 ^/L, M (Q1, Q3)]	4.36 (3.3,5.8)	4.26 (3.26,5.57)	4.5 (3.56,6.39)	0.052
lymphocyte, [×10^9 ^/L, M (Q1, Q3)]	1.53 (1.15,1.95)	1.6 (1.2,2.02)	1.4 (1.07,1.8)	0.002
monocyte, [×10^9 ^/L, M (Q1, Q3)]	0.45 (0.37,0.6)	0.44 (0.37,0.59)	0.5 (0.37,0.62)	0.137
hemoglobin, [g/L, M (Q1, Q3)]	135 (122,145)	138 (126,147)	127 (118,138)	<0.001
Blood platelet, [×10^9 ^/L, M(Q1,Q3)]	203 (168,236)	202.5 (171.25,232.75)	207 (153,243)	0.696
Hs-CRP, [mg/L, M (Q1, Q3)]	2.56 (0.64,7.65)	2.31 (0.61,6.36)	4 (0.73,13.91)	0.006
Prothrombin time, [sec, M (Q1, Q3)]	11.2 (10.8,11.8)	11.2 (10.7,11.7)	11.4 (10.8,12)	0.015
APTT, [sec, M (Q1, Q3)]	31.4 (29.35,33.2)	31.5 (29.42,33.2)	31.2 (29.1,32.8)	0.239
fibrinogen, [g/L, M (Q1, Q3)]	3.21 (2.84,3.76)	3.17 (2.81,3.69)	3.39 (2.93,3.95)	0.016
D-Dimer, [ng/ml, M (Q1, Q3)]	111 (68,188.5)	97.5 (62.25,156.96)	164 (98,245)	<0.001
Potassium, [mmol/L, M (Q1, Q3)]	4 (3.8,4.2)	4 (3.8,4.2)	4 (3.8,4.3)	0.227
Sodium, [mmol/L, M (Q1, Q3)]	140 (138,142)	140 (138.4,142)	140 (138,141)	0.032
AST, [U/L, M (Q1, Q3)]	21 (16.45,29.2)	21 (17,29)	21 (16,30)	0.471
ALT, [U/L, M (Q1, Q3)]	19 (14,27)	19 (15,27)	17.8 (12,25)	0.021
creatinine, [μmol/L,M(Q1,Q3)]	74 (63.6,88.5)	72 (62,85)	80 (68,100)	<0.001
Uric acid,[μmol/L,M(Q1,Q3)]	334 (280.74,403.5)	326.5 (280.47,394.75)	355 (284,433)	0.047
albumin, [g/L, M (Q1, Q3)]	42 (39.6,44.15)	42.6 (40.3,44.6)	40.2 (38.1,42.6)	<0.001
myoglobin, [ng/ml, M (Q1, Q3)]	52 (34,86)	46.8 (29.25,69)	74.62 (47,151)	<0.001
CK-MB, [ng/ml, M (Q1, Q3)]	2.01 (2,4.4)	2 (2,3.27)	2.97 (2,15)	<0.001
Troponin, [ng/ml, M (Q1, Q3)]	0.02 (0.01,0.81)	0.01 (0.01,0.64)	0.07 (0.01,1.1)	0.007
PNI, M (Q1, Q3)	49.9 (46.33,53.2)	50.65 (47.19,54.15)	47.6 (44.75,51.05)	<0.001
Total cholesterol, [mmol/L, M (Q1, Q3)]	3.85 (3.26,4.63)	3.99 (3.37,4.74)	3.59 (3.07,4.42)	0.003
triglyceride, [mmol/L, M (Q1, Q3)]	1.39 (0.99,1.89)	1.42 (1.03,1.93)	1.31 (0.95,1.8)	0.061
HDL-C, [mmol/L, M (Q1, Q3)]	1.04 (0.9,1.2)	1.04 (0.9,1.2)	1.04 (0.89,1.18)	0.536
LDL-C, [mmol/L, M (Q1, Q3)]	2.09 (1.65,2.65)	2.16 (1.67,2.7)	1.9 (1.6,2.55)	0.015
BNP, M (Q1, Q3)	75.9 (32.85,283.85)	62.2 (25.7,176.6)	228.4 (71.6,491)	<0.001
LVEF, M (Q1, Q3)	58(54,61)	59(57,62)	55(50,59)	<0.001
GRACE, M (Q1, Q3)	99(86.5,113)	96.5(83,106.75)	110(97,126)	<0.001

MACE, major adverse cardiovascular event; NSTEMI, non-ST-segment elevation myocardial infarction; STEMI, ST-segment elevation myocardial infarction; SBP, systolic blood pressure; DBP, diastolic blood pressure; BMI, body mass index; Hs-CRP, hypersensitive C-reactive protein; APTT, activated partial thromboplastin time; AST, aspartate Transaminase; ALT, alanine aminotransferase; CK-MB, creatine kinase isoenzymes; PNI, prognostic nutritional index; HDL-C, high-density lipoprotein-cholesterol; LDL-C, low-density lipoprotein-cholesterol; BNP, brain natriuretic peptide; LVEF, left ventricular ejection fraction; GRACE, global registry of acute coronary events.

### Screening for characteristic variables

The recursive feature elimination algorithm is a model-based method for selecting features. It refines the optimal feature set by continuously training the model and sequentially eliminating features with lower weights. In contrast, Lasso regression introduces the L1 regularization term, which enforces sparsity by constraining the sum of absolute values of the parameter vectors. This allows Lasso to select features and reduce many parameters to zero, simplifying the model structure and improving interpretability. The study applies a recursive feature rejection algorithm combined with the Lasso regression technique to reduce the dimensions of the training dataset. The aim is to filter out parameters that are highly correlated with MACE.

**Figure 1:**
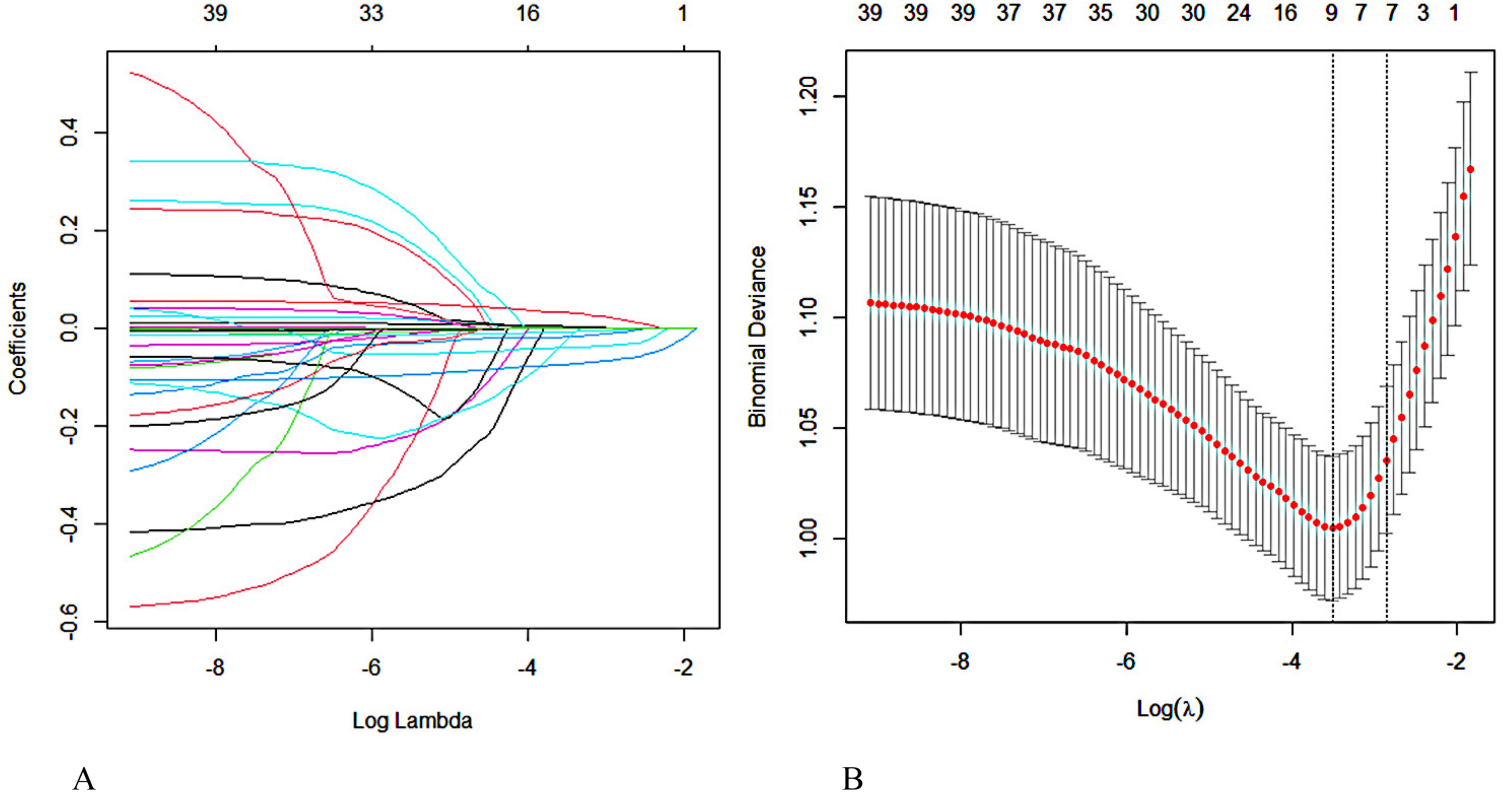
Screening for predictors of MACE within one year after PCI in elderly patients with ACS using lasso regression. **(A)** Regression coefficient variation curve with Log Lambda. **(B)** Obtain the optimal λ process through iterative analysis using the 10-fold cross-validation method.

Figure 1A shows the process of streamlining variables and adjusting coefficients in the LASSO regression model. The upper part of the graph shows the number of variables required by the model, which is gradually reduced from left to right on the horizontal axis, while the lower horizontal axis shows the logarithmic form of the penalty coefficients. The vertical axis reflects the magnitude of the coefficient of each variable in the model. The figure displays the variable importance order in the model. Each colored line represents a different variable. As the penalty increases, the coefficients of less important variables quickly approach zero, while more central variables have coefficients that move less under the penalty term, allowing them to remain until the end. Figure 1B shows the trend of the model's mean squared error with Log(λ), providing a basis for screening the optimal model. The vertical axis uses mean square error (MSE) as a metric to measure the deviation of the model's predicted value from the true value. Each MSE data point has an error bar indicating its 95% confidence interval, enhancing the data's reliability. The figure shows two dotted lines, each with a unique meaning. The left dotted line, lambda. Min marks the horizontal coordinate where the MSE reaches its minimum value, which is a key reference point for optimizing model performance. The right dotted line, lambda.1se represents the distance from lambda. Min by one standard error. This corresponds to a more concise and efficient model since it covers fewer variables. We constructed the model using a rigorous 10-fold cross-validation method for iterative analysis. The model achieved excellent performance and streamlined variables, as shown in Figure 1B when the average error reached its lowest point (λmin = 0.030). This approach effectively avoids overfitting while maintaining high performance, demonstrating its potential for practical applications.

**Figure 2:**
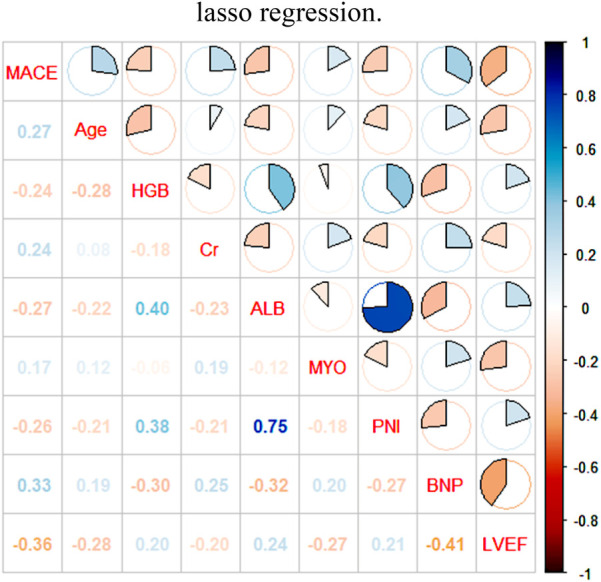
Pearson analysis of MACE in ACS in the elderly and its influencing factors. LVEF, left ventricular ejection fraction; PNI, prognostic nutritional index; BNP, brain natriuretic peptide; ALB, albumin, MYO, myoglobin; Cr, creatinine; HGB, hemoglobin.

After conducting extensive calculations using the recursive feature elimination algorithm, we discovered that the model's accuracy increases to 0.7543 when six variables are incorporated. This accuracy further improves to 0.7629 when eight variables are used, but slightly decreases to 0.7610 when ten variables are included. After conducting a joint evaluation of the recursive elimination algorithm and Lasso regression, we identified eight feature variables: Age, LVEF, myoglobin, Brain Natriuretic Peptide, albumin, creatinine, PNI, and hemoglobin. Figure 2 displays the correlation analysis between eight characteristic variables and the occurrence of MACE in elderly ACS patients. The correlation between LVEF and MACE is the most significant, while the association between myoglobin and MACE is the weakest. Notably, LVEF, PNI, albumin, and hemoglobin are protective factors, and higher values correspond to lower MACE risk. Conversely, the remaining four indicators exhibit the opposite trend.

### Clinical prediction modeling

**Figure 3:**
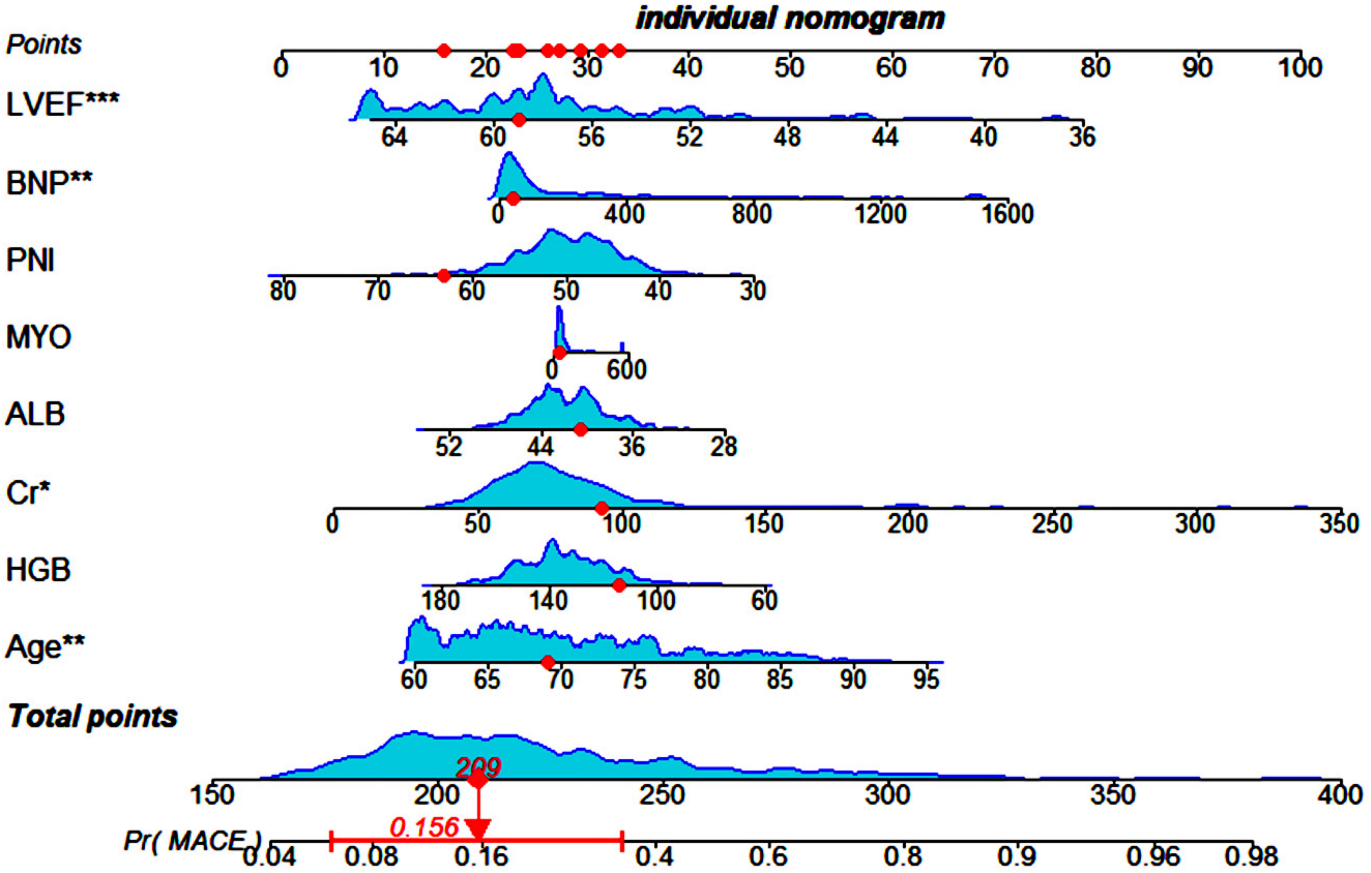
Nomogram depicting the clinical prediction model for the occurrence of MACE within one year after PCI in elderly patients with ACS. LVEF, left ventricular ejection fraction; PNI, prognostic nutritional index; BNP, brain natriuretic peptide; ALB, albumin, MYO, myoglobin; Cr, creatinine; HGB, hemoglobin.

The dependent variable for this study was the occurrence of a MACE event within one year after PCI in elderly ACS patients. The independent variables were carefully selected using the characteristic recursive culling algorithm in conjunction with Lasso regression and consisted of eight predictors. We used multifactorial logistic regression analysis to create a nomogram of the clinical prediction model for MACE within one year after PCI in elderly ACS patients. The diagram is shown in Figure 3. Following the Hosmer-Lemeshow test, we obtained a statistic of *χ*^2^ = 6.245 and a corresponding *p*-value of 0.620. These results strongly confirm the high goodness-of-fit of the predictive model. Additionally, the Brier score of the clinical prediction model we constructed was 0.1502, further confirming its accuracy. We assigned scores to different tiers of values for each indicator based on their weight on the outcome variables in the model. These scores were then combined to obtain a composite score. The predicted value of possible future adverse outcomes for the patient was derived by using the functional correspondence between the composite score and the probability of MACE. Figure 3 shows that the probability of MACE stabilized at 0.156 with a 95% confidence interval range of (0.0612, 0.345) when the composite score reached 209 points. Upon further observation, it was found that the values of LVEF, PNI, albumin, and hemoglobin had a negative correlation with the scores. This means that the scores decreased as the values increased. Conversely, the other four indicators showed the opposite trend, where an increase in the values led to an increase in the scores.

### Comparison of clinical predictive modelling and GRACE scores

The NRI is a valuable tool for evaluating the accuracy of a predictive model. A positive NRI value indicates that the new model outperforms the old model, while a negative NRI value indicates that the new model underperforms the old model. The IDI assesses the change in the difference in predictive probability between two models. This is based on the predictive probability of the disease model for each individual. A larger IDI value indicates better predictive ability of the new model. If the IDI is positive, it indicates an improvement in predictive ability. Conversely, a negative IDI suggests a decrease in predictive ability, while an IDI of zero indicates no improvement in the new model compared to the old model. The NRI covers the net reclassification of all data, while the NRI + and NRI− are net reclassification indices for data with and without MACE, respectively.

**Figure 4:**
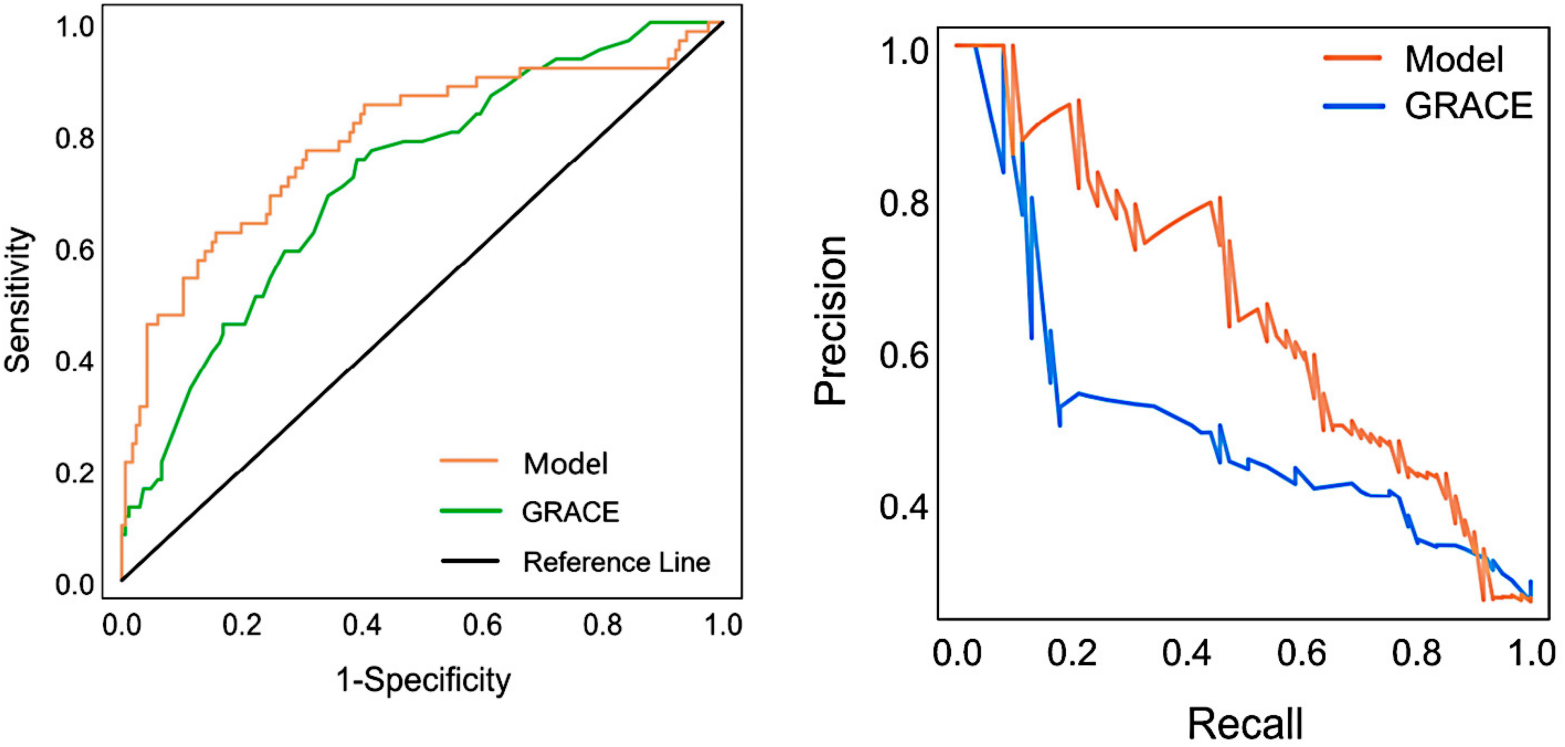
ROC and recall curves are used to evaluate clinical prediction models and GRACE scores.

The study results indicate that our newly constructed prediction model for the studied population outperformed the traditional GRACE scoring system. The NRI value was 0.6167, with NRI + at 0.2263 and NRI− at 0.3903. The IDI was high at 0.1272, and the 95% confidence intervals did not cover the 0 points. The *P*-value was significantly less than 0.001. Please refer to Table 2 for detailed data. To assess the clinical prediction model's performance in terms of differentiation and recall, we plotted the ROC and recall curves (Figure 4). The figure shows that our clinical prediction model has a significantly larger area under the ROC curve and the recall curve compared to the GRACE scoring system. Specifically, the area under the curve (AUC) for the training set reached 0.790, with a 95% confidence interval (CI) ranging from 0.745 to 0.836. In the validation set, the AUC was 0.787, with a 95% CI ranging from 0.714 to 0.861. In comparison, the GRACE score's AUC was 0.720, with a 95% CI ranging from 0.647 to 0.793. All results demonstrated high statistical significance, with *P*-values < 0.001.

**Table 2:** Comparison of clinical prediction models and GRACE scores.

Metric	Estimate	SE	95%CI	*P* value
NRI	0.6167	0.1335	0.3559–0.8807	<0.001
NRI+	0.2263	0.0800	0.0845–0.3984	<0.001
NRI−	0.3903	0.0826	0.2250–0.5543	<0.001
IDI	0.1272	NA	0.0904–0.1641	<0.001

### Validation of clinical prediction models

**Figure 5:**
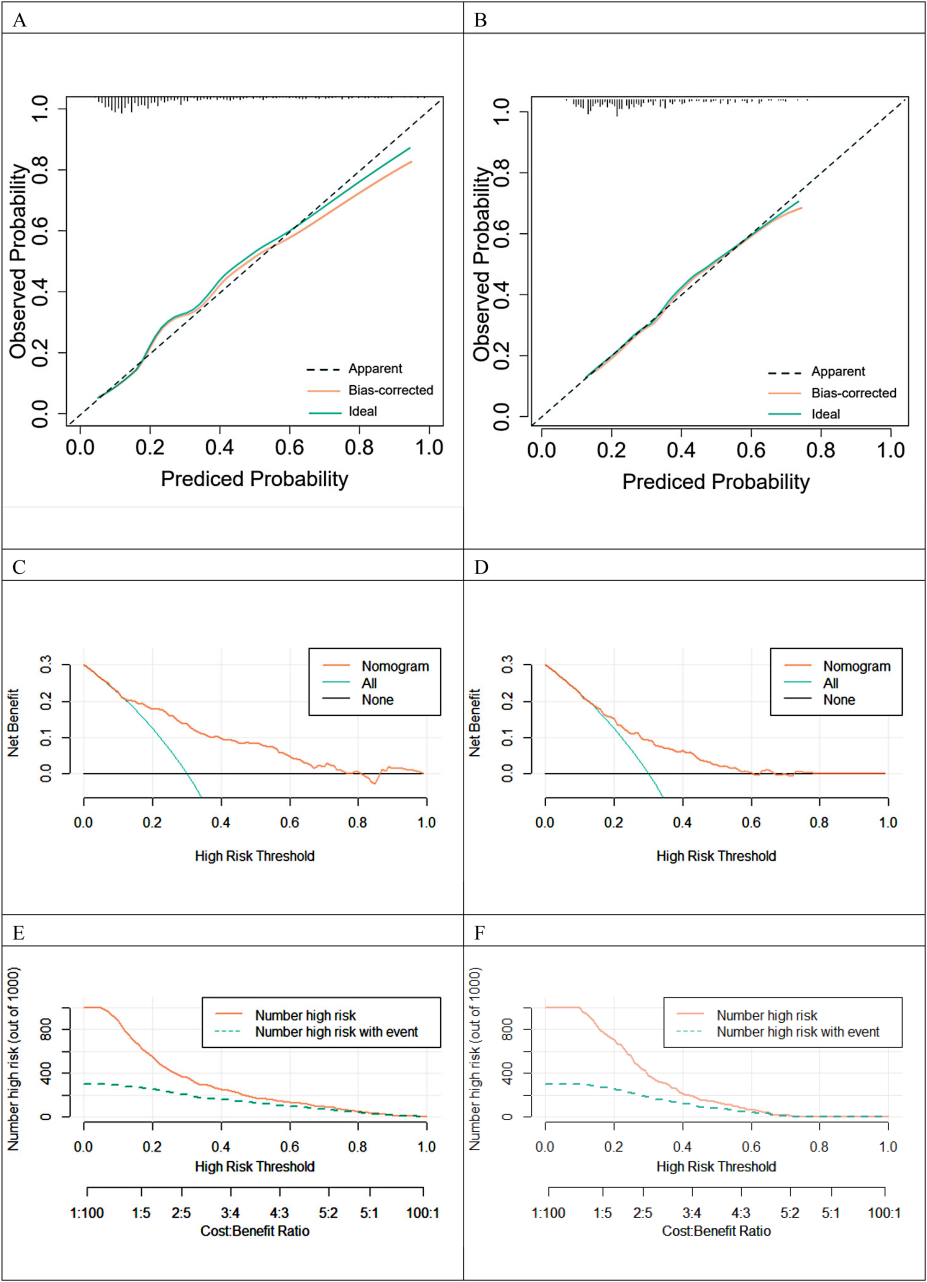
Standard curves, DCA curves, and clinical impact curves are used in clinical prediction models. **(A)** Training Set. **(B)** Validation set. **(C)** Training Set. **(D)** Validation set. **(E)** Training Set. **(F)** Validation set.

The validation set consisted of 227 cases, and the constructed clinical prediction models were rigorously validated. We evaluated the predictive performance of the models in terms of calibration accuracy and clinical impact across multiple dimensions. The graph of the calibration curve shows the predicted probability values on the horizontal axis and the actual observed probabilities on the vertical axis. Figures 5A,B demonstrates a high degree of agreement between the predicted and actual probabilities, indicating the model's excellent correction ability. We use the Decision Analysis Curve (DCA) to explore the model's performance under different risk thresholds (13). The DCA plot shows the net return of the model under each risk threshold, depicted by the red line segment, which follows a smooth trend. The black line segment is parallel to the horizontal axis and has a vertical coordinate of 0. It represents the scenario where all the samples are negative, resulting in a net return of zero as no intervention is taken. The green line segment represents the net return that can be obtained by this sample in this model in the scenario where all the samples are positive. Figures 5C,D shows that the model's net gain performs well when the risk threshold exceeds 0.15, indicating its high validity for clinical applications. The clinical impact curve (CIC) plots the number of misdiagnoses against the risk threshold. The red line represents the number of people designated as high risk in the model, while the green line represents the number of people with a positive outcome. The misdiagnosis rate decreases as the risk threshold increases. When the risk threshold exceeds 60%, the positive estimate closely matches the actual number of people with the disease (refer to Figures 5E,F).

## Discussion

The objective of this study was to develop a clinical risk prediction model for hazard stratification in elderly patients undergoing PCI for acute coronary syndrome (ACS). The high incidence of MACE not only threatens patients’ lives but also imposes a significant economic burden. As the number of elderly ACS patients continues to grow, improving treatment outcomes has become a pressing priority. Building such a model is essential for identifying high-risk patients and implementing timely, effective interventions. Nomograms serve as valuable visualization tools, converting complex regression equations into intuitive, easy-to-read graphs. This enhances the clarity and accessibility of prediction results, enabling clinicians to rapidly assess a patient's condition. By providing precise, individualized survival or risk probabilities, Nomograms align with the principles of precision medicine. They empower clinicians to make informed, patient-specific decisions, ultimately improving care outcomes and optimizing resource allocation.

The recursive elimination algorithm combined with Lasso regression was used in this study to evaluate the training set data comprehensively. From the 44 variables, 8 key predictors were selected as independent variables. Using these variables, a Nomogram prediction model was constructed for MACE events that occur within one year after undergoing PCI in elderly ACS patients. The study found that four indicators—LVEF, PNI, albumin, and hemoglobin—have a protective effect, with an increase in their values predicting a decrease in the risk of MACE. Conversely, the remaining four indicators showed the opposite trend. The Hosmer-Lemeshow test yielded a statistic of *χ*^2^ = 5.247 and *P* = 0.731, indicating excellent goodness of fit for the prediction model. Additionally, the clinical prediction model achieved a Brier score of 0.1502, further confirming its accuracy. The validation set's calibration curves show a strong correlation between predicted and actual probabilities, demonstrating the model's excellent corrective ability. Additionally, the AUC value of the validation set is 0.787, indicating good performance in both differentiation and discriminative ability. Finally, the decision analysis curve and clinical impact curve of the validation set both showed satisfactory net benefit values, indicating strong support for practical applications.

Previous studies have shown a significant link between a low left ventricular ejection fraction (LVEF) and the onset of acute coronary syndrome (ACS) (14). Left ventricular ejection fraction (LVEF) is a core parameter used to assess cardiac function and diagnose heart failure (15). According to an in-depth comparative analysis of 1,429 patients undergoing PCI by Hanada et al., an LVEF of less than 40% strongly predicts MACE in the long term (16). The study confirmed that increasing LVEF can effectively reduce the probability of MACE by curtailing ventricular remodeling (17). The study by Zhang Kangping's team demonstrated that factors such as systolic and diastolic blood pressure, urea levels, the HbA1c/ApoA1 ratio, and D-dimer concentration play a crucial role in predicting in-hospital MACE caused by STEMI in patients undergoing PCI. An individualized Nomogram was developed using these indicators to predict MACE in STEMI patients. The model's discrimination, predictive accuracy, and clinical utility were evaluated and showed excellent significance, with similarly strong performance observed in the validation cohort (18).

PNI is a nutritional assessment method that uses serum albumin concentration and lymphocyte count. It was originally designed to evaluate the immune and nutritional status of patients undergoing gastrointestinal surgery (19). Since then, it has been applied to other disease areas, including cancer, chronic kidney disease, and cardiovascular disease (20). Research has demonstrated a strong correlation between these metrics and prognostic outcomes in patients with acute coronary syndrome (ACS) (6). In cases of ACS and stable coronary artery disease (including previous myocardial infarction and heart failure), low levels of albumin are of prognostic value (21). Additional studies have suggested that the low albumin phenomenon in stable CAD may be triggered by systemic atherosclerotic inflammation (22). Previous studies have shown that advanced age and high creatinine levels are significant risk factors for MACE in ACS patients undergoing PCI (23, 24). Additionally, reduced hemoglobin levels may increase the risk of bleeding, which can exacerbate the likelihood of MACE in patients with ACS (25, 26).

In conclusion, this study developed a clinical prediction model for MACE in elderly ACS patients who underwent PCI. The risk probability of MACE can be visualized using a nomogram. However, this study has several limitations, including being a single-center retrospective study. Therefore, its results need further validation using large-sample, multicentre data. The performance of the nomogram prediction model may deteriorate over time due to changing risk factors of the disease, unmeasured risk factors, therapeutic measures, therapeutic background, patient attention, adherence, psychological factors, etc. We encourage other researchers and medical centers to contribute to and enhance this study.

## Data Availability

The original contributions presented in the study are included in the article/Supplementary Material, further inquiries can be directed to the corresponding author.
